# Spectral Components of Honey Bee Sound Signals Recorded Inside and Outside the Beehive: An Explainable Machine Learning Approach to Diurnal Pattern Recognition

**DOI:** 10.3390/s25144424

**Published:** 2025-07-16

**Authors:** Piotr Książek, Urszula Libal, Aleksandra Król-Nowak

**Affiliations:** 1 Department of Acoustics, Multimedia and Signal Processing, Wroclaw University of Science and Technology, 50-370 Wroclaw, Poland; 2 Department of Mechanics and Vibroacoustics, AGH University of Krakow, 30-059 Krakow, Poland

**Keywords:** honey bee sound, acoustic sensing, beehive acoustic monitoring, machine learning, precision apiculture, spectral analysis

## Abstract

**Highlights:**

**What are the main findings?**

- Near-perfect classification of time-of-day categorized honey bee sounds is possible using both deep learning methods and classical machine learning.
- The most important frequency bands for categorization of honey bee sound time-of-day are between 100 and 600 Hz, in any case not exceeding 2 kHz.

**What is the implication of the main finding?**

- Complex and computational costly models may not be required in certain cases for analyzing honey bee sounds.
- Data collection of honey bee sound signals for AI-based honey bee diurnal pattern monitoring may be performed with sampling rates as low as 4 kHz.

**Abstract:**

This study investigates the impact of microphone placement on honey bee audio monitoring for time-of-day classification, a key step toward automated activity monitoring and anomaly detection. Recognizing the time-dependent nature of bee behavior, we aimed to establish a baseline diurnal pattern recognition method. A custom apparatus enabled simultaneous audio acquisition from internal (brood frame, protected from propolization) and external hive locations. Sound signals were preprocessed using Power Spectral Density (PSD). Extra Trees and Convolutional Neural Network (CNN) classifiers were trained to identify diurnal activity patterns. Analysis focused on feature importance, particularly spectral characteristics. Interestingly, Extra Trees performance varied significantly. While achieving near-perfect accuracy (98–99%) with internal recordings, its accuracy was considerably lower (61–72%) with external recordings, even lower than CNNs trained on the same data (76–87%). Further investigation using Extra Trees and feature selection methods using Mean Decrease Impurity (MDI) and Recursive Feature Elimination with Cross-Validation (RFECV) revealed the importance of the 100–600 Hz band, with peaks around 100 Hz and 300 Hz. These findings inform future monitoring setups, suggesting potential for reduced sampling frequencies and underlining the need for monitoring of sound inside the beehive in order to validate methods being tested.

## 1. Introduction

Beekeeping is a vital part of the agricultural industry, providing a wide range of bee products such as honey, wax, and propolis. These products are used in diverse industries, ranging from mounting accelerometers with beeswax for machine vibration measurement to incorporating propolis in pharmaceutical products. Furthermore, bee activity is essential to many ecosystems containing insect-pollinated plants, as bees are important pollinators. Scientific research concerning bee behavior and the effects on their activity has been ongoing for decades, with much of this research focused on profit maximization in industrial apiaries.

A significant concern in modern beekeeping is monitoring bee colony health and productivity. Traditional beekeeping techniques involve manual inspections, which require opening hives and visually inspecting the honeycombs. Such inspections are stressful for the colony and require a substantial investment of labor and equipment. This investment is necessary, especially to prevent diseases and parasites, which negatively impact both colony health and productivity. Adequate monitoring helps beekeepers to prevent these unwanted outcomes, and automated electronic monitoring systems can reduce costs and mitigate the negative impacts of standard hive inspections.

Research on automated honey bee monitoring systems focuses primarily on parameter selection and the effectiveness of various analysis methods. Studies often investigate well-established parameters such as temperature and humidity [1,2]. Beehive weight—strictly correlated with honey production in the beehive—is another commonly used parameter in manual colony assessments [3]. Monitoring these parameters in conjunction with manual hive inspections enables beekeepers to proactively manage their colonies. For example, a beekeeper might initiate supplemental feeding earlier in late summer if a weakened colony struggles to produce sufficient food stores for winter survival [4].

However, indicators of colony health are not limited to these parameters. Experienced beekeepers also consider bee sounds, often intuitively estimating the mood of the colony before initiating a manual assessment. While many studies utilize accelerometers, which are easier to mount within hives due to reduced risk of propolization and beeswax buildup on sensors [5,6,7], some research has explored audio monitoring using microphones. As work in the field has been ongoing for multiple decades, the types of transducers and the possibility for injecting them in different locations around the bee colony have evolved. In the beginning, simple tube-based devices were used, such as the Woods Apidictor [8]. With the advent of compact microphones and solid-state amplification, the possibilities for recording honey bee sounds have greatly widened.

In a typical case, microphones are currently mounted either inside or outside the hive; however, standardized microphone placement and internal microphone protective enclosure designs have not been established. Some experiments have focused on different methods of internal monitoring employing both accelerometers and microphones, while others have looked at external microphone placements [9]. Many publicly available datasets of honey bee sounds are present in open access repositories such as the MSPB dataset, which is a semi-longitudinal study of internal honey bee sounds [10]. Citizen science can also produce datasets for monitoring honey bees. One example is the Nu-hive dataset connected to the Open Source Beehives project (OSBH), which consists of external sound recordings collected by various beekeepers [11]. More specialized datasets connected with specific experiments are also available, such as the work by Rustam et al. [12]. Despite the large amount of both sound and parametric data in these datasets, none contain comparable recordings taken both inside and outside beehives. As such, a comprehensive comparison of the results obtained using internal and external microphones for simultaneous recording in both locations is lacking, and there is no consensus on the optimal placement of the microphone for honey bee monitoring.

Many studies have introduced single-purpose measurement setups without comparing microphone mounting locations. Examples include swarming prediction based on external microphones [13] and detecting the presence of the queen bee from audio recorded inside the beehive on a low-power microcontroller [14]. Results are then analyzed within the context of the specific task presented. Existing measurement setups have utilized sensors mounted at the bottom of the hive [15] or within the walls of its superstructure [16]. In both cases, internally mounted microphones require protection from propolization and beeswax accumulation as well as strategic positioning to ensure unobstructed sound access from multiple directions. Protection is typically achieved by using a specialized enclosure or a metal mesh to separate the bees from the sensor. The type of beehive needs to be taken into account when developing measurement systems; for example, methods possible in Langstroth-type beehives may not be available in horizontal beehives. Consequently, each experiment involves distinct considerations for microphone protection, hindering comparison between methods. Microphones are sometimes mounted outside the beehive; an example of such practice is the work by Kulyukin et al. [17], where microphones were mounted approximately 10 cm above the beehive landing pad. However, no research to date has shown a direct comparison of results obtained while using microphones mounted both inside and outside the beehive.

A recent trend involves moving beyond audio monitoring to utilize radar sensors, as proposed in studies on detection of swarming and robbing activity [18] and automated classification of incoming and outgoing honey bees [19,20]. However, these radar applications primarily focus on the flight patterns of bees outside the hive, and seek to provide alerts about external conditions rather than the colony’s internal state and mood.

While some studies have compared different analysis methods for bee sound monitoring data, often focusing on machine learning techniques and hyperparameter tuning (e.g., [5]), and others have compared groups of methods (e.g., comparison of machine learning and deep learning approaches [17]), these works do not compare results obtained using different microphone locations. Although Kulyukin et al. [17] suggested their methods are applicable to both internal and external recordings, no direct comparison was performed, and it remains unclear whether both locations yield comparable results.

Diurnal patterns in honey bee activity have been known to exist for decades. Attempts to characterize the circadian rhythm of bees have been made using both standard observation techniques [21] and automated monitoring of beehive weight at the beginning and end of the activity period [22]. Work to correlate bee sound parameters with the time of day has been carried out in the past, with Cejrowski et al. [23] showing clear daily activity peaks in recorded signal energy. Prakhar Amlathe [24] also tackled the problem in his thesis, where classic machine learning techniques were compared with deep learning with time-of-day classification used as the test problem.

In this study, we employ simultaneous audio recordings from microphones placed both inside and outside the hive, using the recognition of the time of day as a classification problem for a honey bee abnormal state detection system. This work aims to demonstrate that in certain cases analyzing externally recorded data can be as effective as using internally recorded data, while also simplifying instrument maintenance. Successful external monitoring would significantly reduce the complexity and cost of automated honey bee monitoring, thereby improving its accessibility. The main contributions of this paper are as follows:

- Analysis of bee activity data across different times of day.
- Design of a protective cage within a brood frame to house an internal microphone and prevent propolization.
- Preprocessing of audio signals acquired from microphones placed inside and outside the beehive using three representations based on Power Spectral Density (PSD).
- Training of Extra Trees and Convolutional Neural Network (CNN) classifiers to identify standard diurnal patterns of honey bee activity.
- Derivation of practical conclusions regarding bee sound analysis and the identification of important spectral features for differentiating bee activity at various times of day, obtained by the feature selection methods of Mean Decrease Impurity (MDI) and Recursive Feature Elimination with Cross-Validation (RFECV) combined with the Extra Trees classifier.

The remainder of this article is organized as follows: Section 2 describes the research methodology and data collection using internal and external microphones; Section 3 presents the experimental results for classifying audio recordings into five time-of-day categories; a discussion of the results is provided in Section 4; finally, Section 5 presents the conclusions.

## 2. Materials and Methods

### 2.1. Data Collection

The measurement station hardware primarily consisted of readily available off-the-shelf components. This approach aligns with the findings of Kulyukin et al. [17] demonstrating that such devices can provide satisfactory results while minimizing per-unit cost. The design largely follows Kulyukin’s BeePi project [25], with modifications specific to this experiment. To achieve the primary objective, two separate USB sound cards (Axagon ADA-12) were used, both connected to a Raspberry Pi 4 single-board computer. These sound cards offer a 16-bit depth, up to a 48 kHz sampling rate, and a 93 dB signal-to-noise ratio. The microphones connected to the sound cards were Mozos LavMic1 Lavalier Electret microphones, with a specified frequency range of 50 Hz to 20 kHz and a signal-to-noise ratio of 76 dB or greater. The omnidirectional microphones’ small size facilitated mounting both inside and outside the hive. A DHT 22 hygrothermometer was also mounted inside the hive for monitoring purposes, although its data are not directly relevant to this experiment. The measurement station was powered by a mains connection using a manufacturer-provided Raspberry Pi power supply.

**Figure 1:**
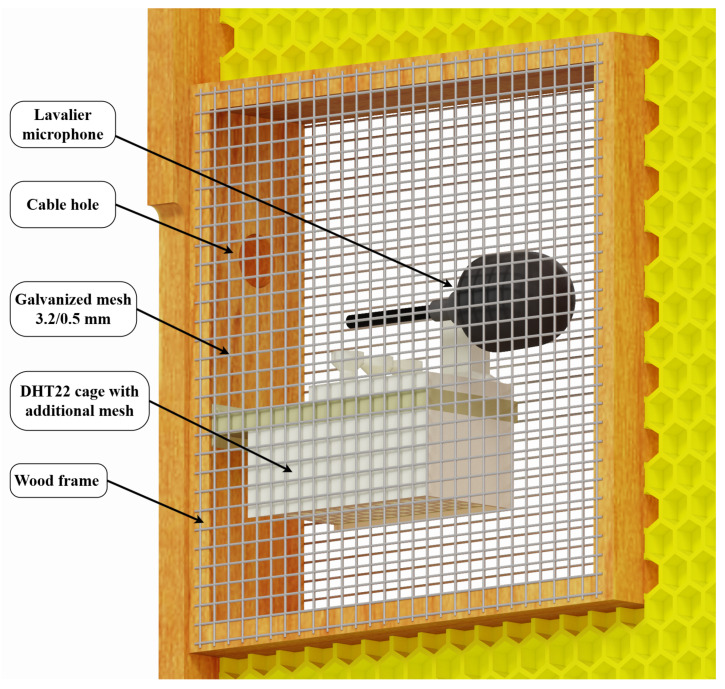
CAD model of protective cage for internal measurements. The cage is designed to be screw-mounted within a Warsaw-type beehive frame.

**Figure 2:**
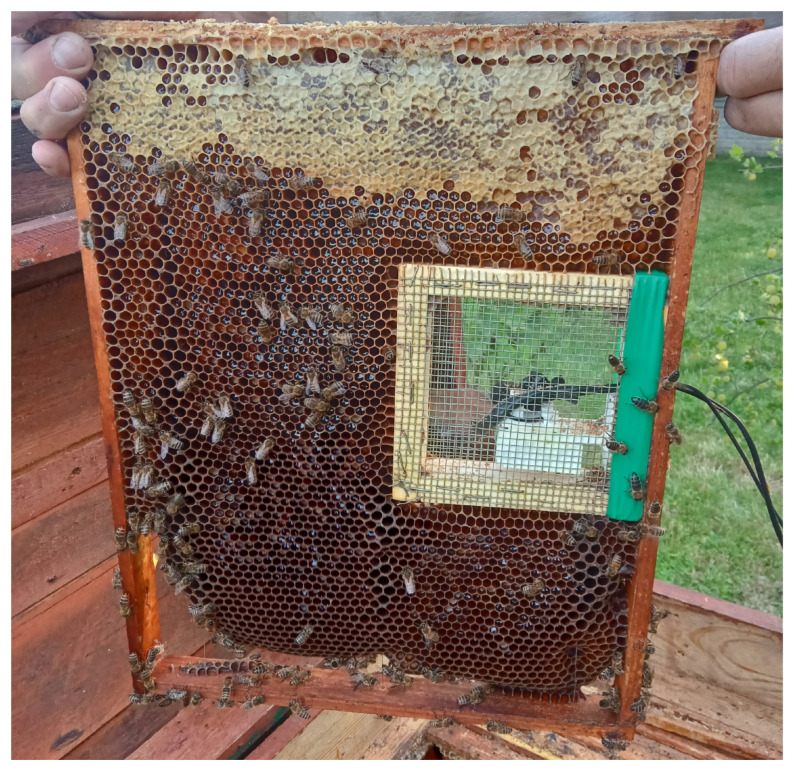
Photo of specialized protective enclosure mounted within a brood frame, protecting microphone from propolization and beeswax buildup. The setup is inserted into a lived-in frame as opposed to a new one in order to reduce the stress caused by foreign objects inside the hive. Photograph taken after measurements.

**Figure 3:**
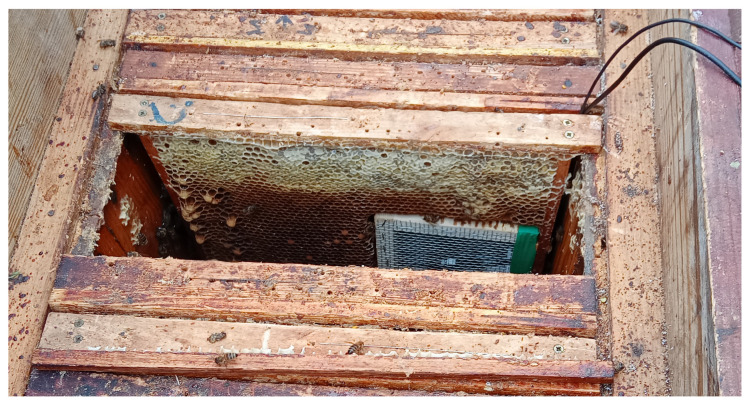
Internal microphone location after mounting the frame within the beehive. Photograph taken at the start of measurements; capped brood can be seen beside the setup.

Due to the dangers of propolization and beeswax buildup on measurement instruments as well as to limit the stress of a foreign body being present within the beehive, a specialized protective cage was developed to achieve successful mounting on the inside of the beehive. The protective cage consisted of a wood enclosure mounted in the middle of a brood frame protected on both sides of the frame with a high-density galvanized mesh with a mesh size of 3.2 mm and a wire diameter of 0.5 mm. Within the enclosure, as shown in Figure 1, a 3D printed microphone mount was implemented to keep the microphone as close to the geometric center of the enclosure as possible, with a vertical pin used to mount the clip of the Lavalier microphone and three tension relievers to keep the microphone in place during mounting. The final location of the internal microphone enclosure, shown in Figure 2 and Figure 3, was chosen in order to minimize the impact of measurement devices on the beehive. The modified brood frame containing the protective enclosure was placed into the beehive with the measurement device on the side of the frame furthest from the entry to the hive.

**Figure 4:**
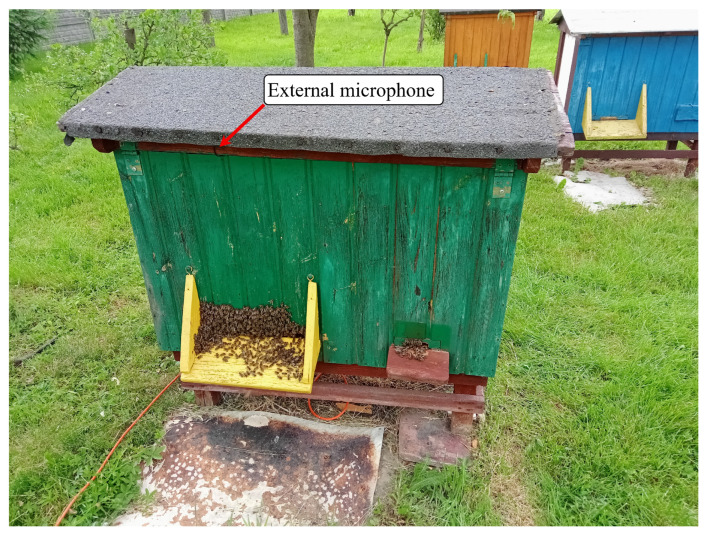
External microphone mounting location outside the Warsaw-type beehive.

The external microphone required no specialized enclosures and was attached to the outside wall of the beehive above the beehive entry using mounting tape. It was protected from rain and environmental factors by the beehive’s roof overhang. The location of the external microphone is shown in Figure 4.

To obtain a test dataset from a single hive, a custom script was developed to manage audio recordings. At the beginning of each minute, the script initiated two simultaneous recording threads, each capturing data from one microphone. Both threads recorded for 50 s, allowing 10 s for compression and storage on the SD card. Data were recorded using the ALSA arecord program and compressed using the LAME package. MP3 compression was chosen in order to minimize storage requirements, maximize monitoring duration, and because previous research has demonstrated that high-bitrate MP3 compression does not compromise the effectiveness of similar bee sound signal analyses [26]. Each file was named according to the scheme [date and time of recording]-[microphone location] to distinguish between microphone placements and encode the recording timestamp within the filename.

The measurement kit was set up on 18 May 2022 in a Warsaw-type beehive in an orchard surrounded by rapeseed fields and wildflower meadows. The colony in which the measurements were set up was an *Apis mellifera carnica* colony with a one-year-old apparently healthy queen bee. The apiary in which measurements were performed counted four active bee colonies spaced equidistantly at a distance not shorter than three meters. At first, it was mounted for a four-day test period during which no recordings were made and the bee behavior was observed. After no obvious negative reaction to the measurement kit’s presence were noted, sound measurements started. Recordings were created between 22 May and 25 June. During the measurement period, two loss of power incidents occurred. After accounting for the downtime from power loss, the combined up-time of the measurement setup was over 29 days. The resulting dataset contained over 75,500 MP3 files and took up more than 40 GB of hard drive space.

**Figure 5:**
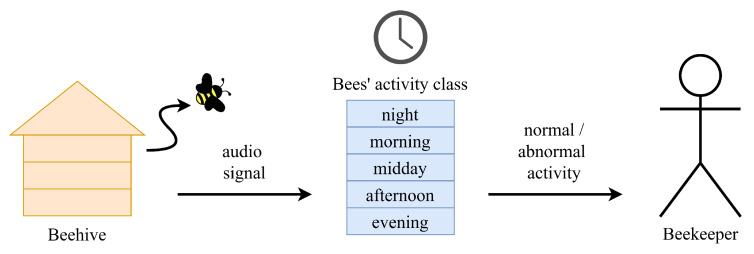
Conceptual honey bee anomalous behavior detection system.

This work is performed in the context of establishing methods for evaluating the state of the honey bee colony to locate anomalous behavior using an automated monitoring system. A monitoring system may use the methods proposed in this work, which are trained on sounds produced by a healthy bee colony, in order to evaluate the state of a given bee colony and detect anomalies based on the error rate of the classification method. Anomalous behavior of honey bees would be a clear indicator of some environmental factors or problems with the colony health. A conceptual system utilizing the methods tested in this work is presented in Figure 5.

### 2.2. Data Analysis Methods

To ensure a fair comparison of data analysis results across different microphone locations (inside or outside the hive), a proven method for analyzing bee sound data was required. Deep learning methods have demonstrated effectiveness and improved generalization on complex datasets without extensive hyperparameter tuning, although they require greater computational resources for training (a factor irrelevant to this experiment). Convolutional Neural Networks (CNNs) were selected due to their ability to overcome limitations of classical machine learning methods and their adaptability to evolving data inputs [27]. Prior to network training, the data underwent preprocessing. First, recordings coinciding with power loss events were removed. Then, recordings from the internal and external microphones were separated and all subsequent processing was performed independently for each microphone location.

The times of sound recording were deciphered from the names of the individual files. Categories corresponding to individual times of day were also determined. The natural times of bee activity were considered in the division. The time-of-day classification problem has been tackled by Prakhar Amlathe in the past [24], and may be useful in future applications concerned with honey bee health monitoring. In our study, we employed the same division into time-of-day categories (classes), which is provided in Table 1.

To reduce the amount of data being processed, 60,000 observations were randomly selected from the entire set. Stratified random sampling was used to select observations, ensuring that the proportion of each class in the sample matched the original dataset, as the length of the times of day used for establishing classification is not uniform. The resulting subset was then divided into a proper training set and a test set in a 4:1 ratio. The division was carried out with stratification. In the next step, feature extraction of the sound signal was performed. The Mel-Frequency Cepstral Coefficients (MFCCs) and Power Spectral Density (PSD) were selected for feature extraction. MFCCs have proven useful in the past [17], and the common use of PSD in noise signal analysis [28] indicates it as a useful feature. Five random samples of 4 s each in length were selected from each processed recording. The samples did not overlap with each other. We extracted 25 MFCC coefficients from each sample and calculated the PSD with a window length of 1024 samples and 256 sample overlap. The data were saved for processing by the convolutional network.

**Figure 6:**
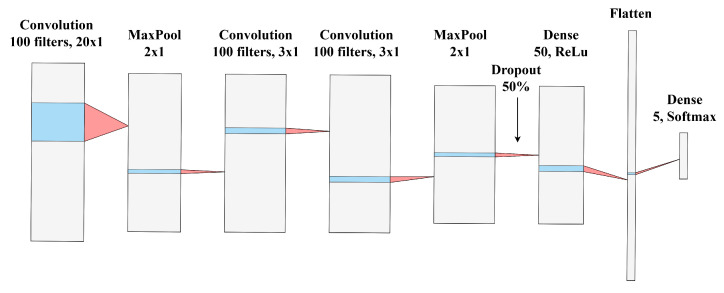
Convolutional neural network architecture used for honey bee activity classification.

A deep learning convolutional network was designed with five-way classification in mind. The network architecture, shown in Figure 6, consists of an input layer, three 2D convolutional layers, two max pooling layers, one dropout layer, and two fully connected layers. The designed output of the network is a five-value vector containing probabilities of the sample under test belonging to every time-of-day category associated with a class listed in Table 1. Four instances of the network were created, each corresponding to a different feature-location combination. All network instances were trained on an NVIDIA RTX 3060 graphics card using the Keras library with a TensorFlow backend. The networks were trained for 15 epochs with a batch size of 128 recordings. The optimization algorithm used in the training was the Adam algorithm. The cost function used was categorical cross-entropy, and the objective function was weighted classification accuracy.

### 2.3. Feature Importance Investigation

While CNNs have achieved promising results, neural networks generally lack explainability, which is often more accessible in classical machine learning methods. To address this issue, a simpler model was required both due to the high computational cost of training CNNs and the potential for a straightforward metric explaining the importance of given features within the dataset. An emphasis was placed on PSD features of the analyzed signals, as they are generally more interpretable than other feature types and because identifying key frequency bands may enhance future monitoring efforts. The Extra Trees meta-estimator, a specific variant of a Random Forest [29] composed of multiple decision trees, was chosen for feature importance analysis. Random Forests are widely used for feature importance because their methodology allows for straightforward extraction of feature significance via the Mean Decrease Impurity (MDI) [30], also known as the Gini importance. However, MDI has known limitations, including sensitivity to correlated features and a bias toward variables with more unique values.

The Extremely Randomized Trees (Extra Trees) method, introduced by Geurts et al. [31], is an ensemble learning technique that constructs multiple decision trees. Unlike traditional Decision Trees or Random Forests, Extra Trees introduces additional randomness by selecting splits completely at random in terms of both features and cut-points while still finding optimal parameter sets. This randomization enhances model diversity and reduces variance, which can improve generalization, particularly in high-dimensional datasets, though at the cost of slightly increased bias.

Specifically, while standard Decision Trees optimize an impurity criterion I(S) (e.g., Gini impurity or entropy) to find the best split *S*:

(1) $$I\left(S\right)=H\left(S\right)-\left(\frac{|S_{L}|}{\left|S\right|}H\left(S_{L}\right)+\frac{|S_{R}|}{\left|S\right|}H\left(S_{R}\right)\right),$$

where $S_{L}$ and $S_{R}$ are the left and right splits and H(S) is the impurity function, Extra Trees selects the split threshold θ randomly from a uniform distribution taken from the range of feature $x_{j}$:

(2) $$\theta ∼U(x_{j}^{\min},x_{j}^{\max}).$$

For classification, a majority voting mechanism is employed:

(3) $$\hat{y}=\arg\max_{c}\sum_{k=1}^{K}⊮\left\{\;f_{k}\left(x\right)=c\right\},$$

where *K* is the number of trees and $f_{k}\left(x\right)$ is the class prediction of the *k*-th tree. This increased randomization contributes to the robustness and generalization ability of the Extra Trees method and their extensions [32] to AI meta-learners.

To investigate the impact of each feature on the classification of time of day, Extra Trees classifiers with 128 trees and a maximum depth of 50 nodes were trained on three different variants of the base PSD dataset. CNNs with unchanged topology were also trained and tested on the data as a reference. No variables were modified except for the dataset in the training of those additional networks. The included variants are as follows:

- PSD—Power spectral density data with the spectrogram window length at 1024 samples and 50% overlap.
- PSD-D—Power spectral density data with the spectrogram window length considerably increased to allow a frequency step of 5 Hz with 50% overlap.
- PSD-D-LC—PSD-D dataset with a fifth-order high-pass Butterworth filter applied with a cutoff frequency of 75 Hz. This dataset was created to cut power grid prime harmonic noise from the analysis as well as to ensure that frequencies below the pass-band of the microphone used in measurement were not considered in the classification.

To verify the MDI results, Recursive Feature Elimination with Cross-Validation (RFECV) [33] was used to select the most relevant features for classification across all additional datasets. This method is more robust to potential high cardinality of datasets and to specific random distributions of training and test data. RFECV was calculated using the same type of Extra Trees classifier as the MDI evaluation, with one key difference: due to the high computational complexity of the method when using a large dataset, the Recursive Feature Elimination was fed a dataset with 10% of the size of the main dataset. RFECV was set up to use cross validation with five folds per step, with a step of ten features.

## 3. Results

**Figure 7:**
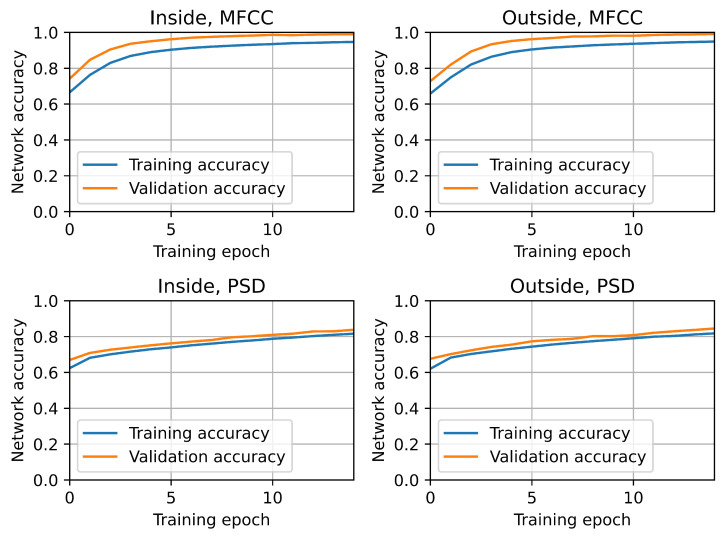
Convolutional network goal function training history.

**Figure 8:**
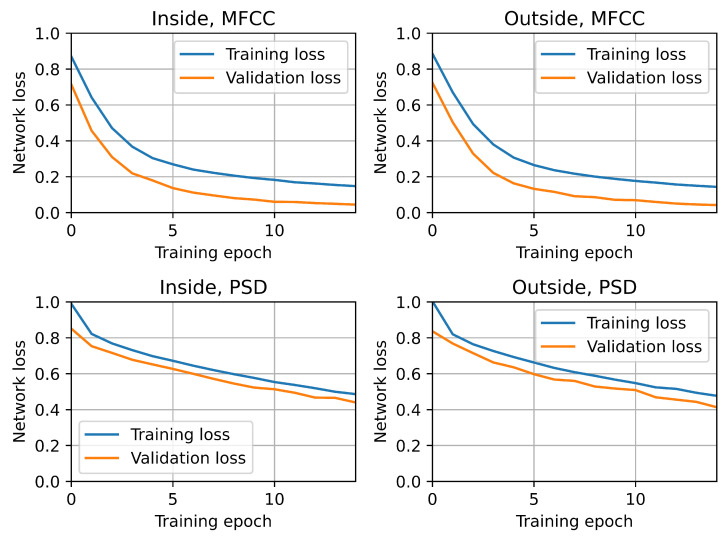
Convolutional network loss function training history.

All trained convolutional neural network models successfully completed training. No obvious overfitting was observed, as both the loss function and the objective function largely maintained their monotonicity. Figure 7 and Figure 8 show that the validation loss was consistently lower than the training loss, while the validation accuracy was consistently higher than the training accuracy. Importantly, these relationships were observed across all CNN model variants, indicating that externally collected samples (from the microphone placed outside the hive) exhibit similar behavior to internally recorded samples.

Table 2 presents a clear comparison of CNN classification results using MFCC and PSD feature representations of audio recorded from both internal and external microphones.

The models trained on MFCC features yield exceptionally high performance, with both internal and external microphones achieving accuracy, F1-score, and recall values exceeding 98%. Notably, the external microphone actually performs slightly better than the internal one across all three metrics. This suggests that for time-of-day classification, MFCCs extracted from externally recorded audio are just as informative, if not slightly more so, than those from internally recorded audio. The near-identical performance indicates that microphone placement has minimal impact when using MFCCs.

The PSD features show considerably lower performance compared to MFCCs. While still reasonable, the accuracy, F1-score, and recall values are in the 76–78% range. Again, the external microphone shows a slight performance advantage over the internal microphone. This suggests that while PSD features can be used for time-of-day classification, they are less discriminative than MFCCs. The difference in performance between internal and external microphones is also small, but still favors the external placement.

The most notable observation is the substantial performance gap between MFCC and PSD features. This highlights the importance of feature selection for this classification task. MFCCs, which are designed to capture the spectral envelope of audio signals and are commonly used in speech recognition, are clearly much better suited for distinguishing different bee activity sounds associated with time of day. PSD, while providing information about the frequency content, may not capture the nuances in the bee sounds as effectively as MFCCs.

Across both feature types, the differences in performance between internal and external microphones are minimal. This suggests that for the task of time-of-day classification, microphone placement (at least in the configuration used in this study) is not a critical factor. The slightly better performance of the external microphone may be due to factors such as reduced interference from hive vibrations or other internal hive noises.

### 3.1. Feature Investigation Results

The experiment using the Extra Trees classifier and the RFECV method provided insights into the most important frequency bands (identified through PSD analysis) for time-of-day classification. Results indicate that frequencies below 1 kHz are predominantly utilized, although specific frequency usage varies across datasets. Table 3 presents the accuracy results of all model variants along with additional validation of the CNN’s performance on these datasets.

The results in Table 3 show that the Extra Trees classifier is not robust against changing the location of the microphone when performing time-of-day classification. The CNN accuracy is very similar between microphone placements, never differing by more than a percentage point. However, the highest accuracy results are achieved by the Extra Trees classifier on internal recordings with values reaching over 99% in the case of the PSD-D and PSD-D-LC datasets.

#### 3.1.1. Extra Trees Importance Metric

**Figure 9:**
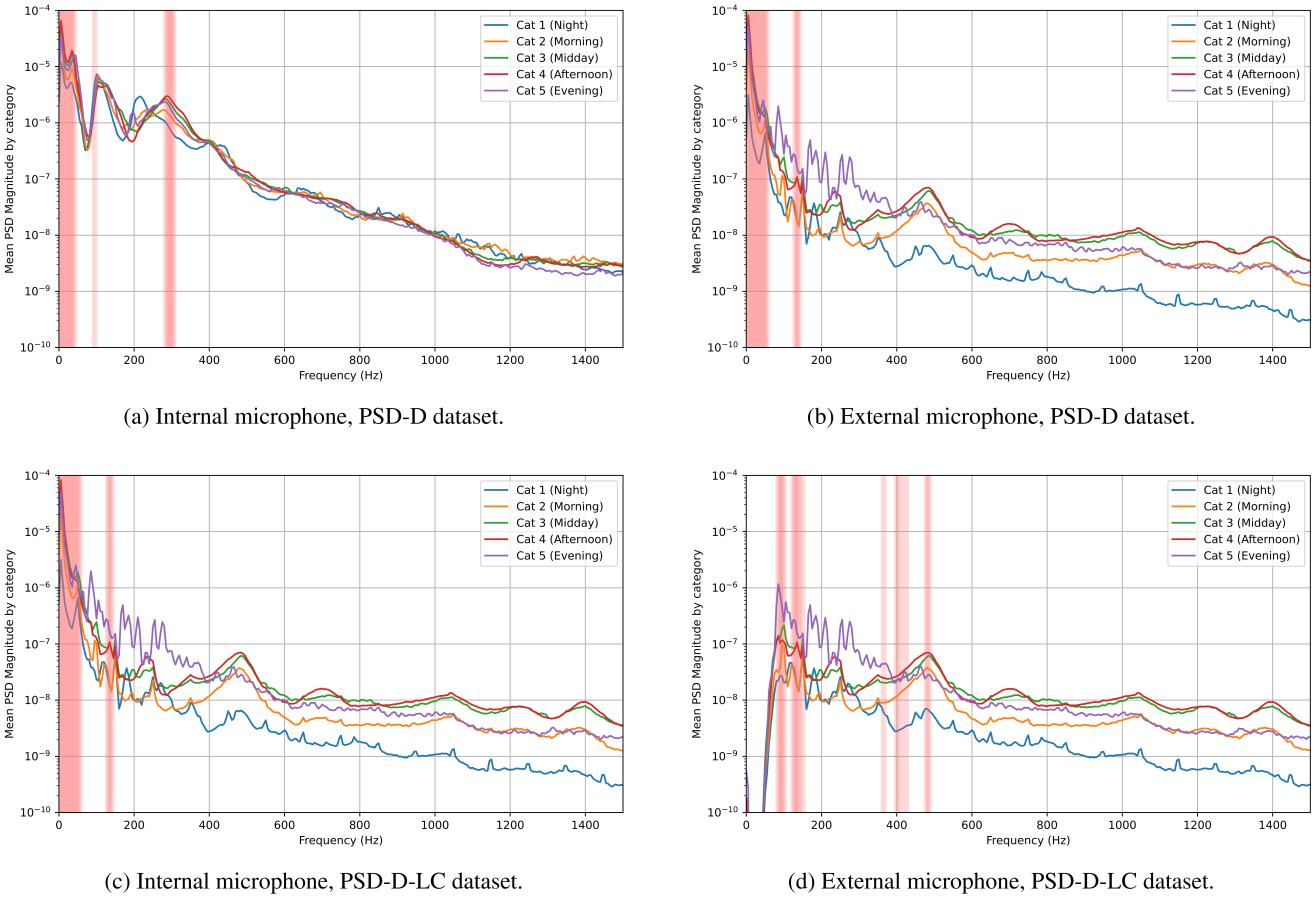
Most important frequencies by Mean Decrease Impurity (MDI) for both tested locations, PSD-D and PSD-D-LC dataset variants.

The Extra Trees importance metric was extracted using the Mean Decrease Impurity method and sorted in decreasing order. Figure 9 shows the categorically divided mean characteristic values, with highlighted lines showing the ten frequencies with the highest MDI score. It can be seen that in the PSD and PSD-D datasets, the most important frequencies for classification are very low. In the case of PSD-D-LC, the most important frequencies are shifted upward to bands around 110–150 Hz, as well as 280–320 Hz for internal sound recordings and around 80–150 Hz and 350–500 Hz for external classification.

#### 3.1.2. RFECV Importance Ranking

**Figure 10:**
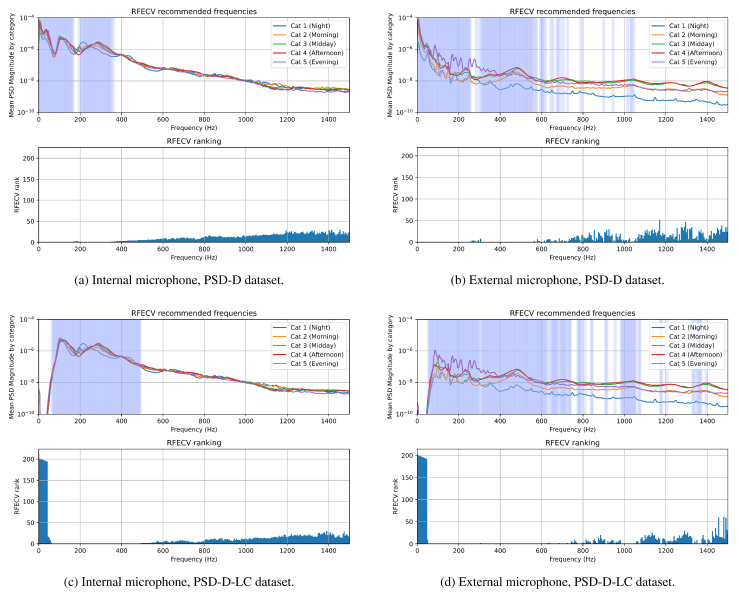
Frequencies recommended by Recursive Feature Elimination with Cross-Validation (RFECV) for both tested locations, PSD-D and PSD-D-LC dataset variants.

The RFECV scores and recommendations are presented in Figure 10. The top subplot highlights the selected frequencies in blue, with the ranking of each frequency shown in the bar plot below. Lower ranks assigned by the method mean that the feature is more important to the classification. It can be noted that the results broadly replicate the behavior seen in the MDI scores. The RFECV method recommends frequencies in the same bands as the Random Forest model for equivalent datasets. In the PSD-D-LC dataset, the rank for frequencies below 75 Hz is very high, which is expected due to the low-cut filtering of the audio signal.

## 4. Discussion

Classification accuracy results acquired by analyzing data in both microphone locations (internal and external) achieved by the CNNs do not differ visibly, as presented in Table 2 and Table 3. A very high accuracy result was achieved by training the convolutional network on MFCCs of recordings taken both outside and inside the beehive. Results achieved by CNNs when classifying the Power Spectral Density of signals are visibly worse than those achieved when using MFCCs; however, the similarity of results achieved by recordings in different locations is preserved. Results achieved via analysis of MFCCs are on par with those shown in the literature, albeit with a different method of analysis [24].

This behavior was not replicated in additional testing performed using the Extra Trees classifier. In that case, the accuracy is markedly lower on both the base PSD dataset and the additional PSD-D and PSD-D-LC datasets, with the results showing around 30% lower accuracy in classifying externally recorded sounds. However, it is important to note that the Extra Trees classifiers basing on internal recordings achieve near-perfect accuracy, much better than CNNs trained on the same datasets. This suggests that simpler methods may be sufficient for analyzing internal honey bee sounds. In analyzing external sounds, more sophisticated methods may be required, as sounds recorded outside of the beehive contain more anthropogenic noise as well as bird songs and the general soundscape around the apiary.

In classifiers trained on the PSD-D dataset, both the MDI score and the RFECV recommendations focus on very-low-frequency components of the signals recorded outside the beehive as the most important for classification purposes. This highlights the need for careful verification of the audio data, as most of the low-frequency components of the recorded signals originate from electrical noise falling below the 50 Hz lower limit of the microphone pass-band. This may indicate that frequencies suggested as important by both the MDI score and the RFECV method in internal monitoring are very focused around the two main bands of interest produced by honey bees, that is, around 100–150 Hz and 300–500 Hz. This is most clearly seen when analyzing the PSD-D-LC dataset, as cutting the low-frequency components of the signal ensures that the classifier learns from honey bee sounds and ignores the noise present within the recording. It is important to note that the efficacy of the Extra Trees classifier for external recordings drops when trained on the PSD-D-LC dataset; however, the equivalent CNN seems to be robust to this phenomenon, showing little change. The likely cause of the CNN’s robustness in this case may be the nature of the convolutional layers used in the network, as they allow the classifier to learn local patterns as well as global ones, a quality which is not present in Decision Tree-based classification methods.

The frequencies identified as the most important by both feature evaluation methods in internally recorded sounds align with the classical understanding of the sounds generated by honey bees. They fall within the natural vibration frequency range of the honey bee thorax [34], with the upper band of interest proposed by the methods encompassing the natural frequencies of honey bee thoraxes. In external recordings, higher frequency bands were also deemed important by the RFECV method, especially the 400–600 Hz range, which typically contains the sound of bees taking off from the beehive.

## 5. Conclusions

The PSD-based methods used in this experiment were chosen in order to investigate the impact of microphone placement on the efficacy of classification methods for determining the time-of-day category using purely audio data, with a focus on understanding the impact of different frequency bands within spectral audio features on classification. On the other hand, preprocessing using MFCC was employed to validate methods used in the literature on the dataset recorded during the experiment. The latter goal was achieved, with Convolutional Neural Networks showing very high accuracy when analyzing MFCC data both inside and outside the beehive. However, MFCCs are a more computationally complex and advanced signal preprocessing method which does not necessarily provide meaningful information about spectral signal components. As such, feature investigation was performed on PSD, which allows for a closer look into the spectral characteristics of the sound signal.

Despite the difficulty of placing microphones inside beehives and the impact of the measurement apparatus on the bee colony, future sound monitoring experiments may consider performing recordings internally using mesh-protected microphones to develop methods using internal recordings, as placing microphones inside the beehive guarantees an increase in the signal-noise-ratio of honey bee signals to non-honeybee noise, and has shown high classification accuracy (98–99%) using the PSD-based Extra Trees classifier. In particular, in this experiment the Extra Trees classifiers struggled with externally recorded sounds, showing a significant accuracy drop (61–62%), while CNNs utilizing PSD remained indifferent to microphone placement despite the much classification lower accuracy (76–86%).

Additional investigation performed using the Extra Trees classifier in combination with extraction of the MDI metric as well as with the RFECV method showed that the most important frequency bands for classifying the time of day based on honey bee buzzing are mostly between 100 and 600 Hz, with a bimodal spectral distribution inside this frequency band. The first important peak is around 100 Hz, while the second is around 300 Hz. The introduction of a high-pass filter allowed the classifiers to focus on these frequency bands, with the CNNs showing more robustness towards removing low-frequency data. These findings are important as a guide for future monitoring setups, which may choose to lower the sampling frequency of recordings to save on computational complexity and data storage costs. Filtering very-low-frequency non-bee-related sounds from the signals to focus on honeybee-produced sounds is recommended as well.

The models trained using data recorded for this experiment may have further use in honey bee health monitoring systems. With additional training on data collected in another beehive that is in a known healthy state, a system comparing the true time of day to the time classified using our model could provide real-time anomalous behavior monitoring, which could prove useful for beekeepers by indicating that a beehive needs to be checked for the presence of illness or other negative factors. However, such a system would require a more varied dataset in order to be truly reliable.

The results presented in this study are promising and highlight the importance of recording sound inside the beehive to verify methods used in interpreting honey bee buzzing sounds. However, the conclusions drawn here are based on a single bee colony in a single season, and as such must be interpreted as a proof-of-concept which still requires a larger-scale dataset with multiple colonies in order to be verified and generalized. Most importantly, simultaneous monitoring of many beehives in many locations needs to be performed in order to rule out the impact of local microclimate and weather conditions on the experimental results. Furthermore, research should be performed on multiple subspecies of honey bees over several years, which would provide a new general dataset that could be used in training models for honey bee health monitoring using diurnal patterns. The impact of microphone type also needs to be addressed in further research, as well as a comparison of results obtained using different recording lengths and compression algorithms in real-life scenarios.

## Figures and Tables

**Table 1:** Division into time-of-day categories (classes) of bee activity.

Category	Time of Day	Hours
1	night	20:00–8:00
2	morning	8:00–10:00
3	midday	10:00–14:00
4	afternoon	14:00–18:00
5	evening	18:00–20:00

**Table 2:** CNN classification results for MFCCs and PSD audio representations from internal and external microphones.

Feature	Location	Accuracy	F1	Recall
MFCC	Inside	98.33%	98.68%	98.33%
	Outside	98.54%	98.83%	98.54%
PSD	Inside	76.19%	78.28%	76.19%
	Outside	77.44%	78.41%	77.44%

**Table 3:** Extra Trees and CNN classification accuracy for modified PSD-based dataset variants.

Dataset	Location	Extra Trees Accuracy	CNN Accuracy
PSD	Inside	98.28%	76.19%
	Outside	61.06%	77.44%
PSD-D	Inside	99.37%	86.49%
	Outside	71.78%	86.34%
PSD-D-LC	Inside	99.23%	87.04%
	Outside	62.04%	86.65%

## Data Availability

Experiment data will be made available upon reasonable request to the authors.

## Abbreviations

The following abbreviations are used in this manuscript:

AI	Artificial Intelligence
CNN	Convolutional Neural Network
MDI	Mean Decrease Impurity
MFCC	Mel-Frequency Cepstral Coefficients
PSD	Power Spectral Density
RFECV	Recursive Feature Elimination with Cross Validation
